# EEG microstate features for schizophrenia classification

**DOI:** 10.1371/journal.pone.0251842

**Published:** 2021-05-14

**Authors:** Kyungwon Kim, Nguyen Thanh Duc, Min Choi, Boreom Lee

**Affiliations:** 1 Department of Biomedical Science and Engineering (BMSE), Institute Integrated Technology (IIT), Gwangju Institute of Science and Technology (GIST), Cheomdan-gwagiro, Gwangju, South Korea; 2 Department of Psychiatry and Biomedical Research Institute, Pusan National University Hospital, Busan, South Korea; 3 Department of Neurology and Neurosurgery, Montreal Neurological Institute, McGill University, Montreal, Canada; 4 McConnel Brain Imaging Center, Montreal Neurological Institute, McGill University, Montreal, Canada; 5 Ludmer Centre for Neuroinformatics and Mental Health, McGill University, Montreal, Canada; McLean Hospital, UNITED STATES

## Abstract

Electroencephalography (EEG) microstate analysis is a method wherein spontaneous EEG activity is segmented at sub-second levels to analyze quasi-stable states. In particular, four archetype microstates and their features are known to reflect changes in brain state in neuropsychiatric diseases. However, previous studies have only reported differences in each microstate feature and have not determined whether microstate features are suitable for schizophrenia classification. Therefore, it is necessary to validate microstate features for schizophrenia classification. Nineteen microstate features, including duration, occurrence, and coverage as well as thirty-one conventional EEG features, including statistical, frequency, and temporal characteristics were obtained from resting-state EEG recordings of 14 patients diagnosed with schizophrenia and from 14 healthy (control) subjects. Machine-learning based multivariate analysis was used to evaluate classification performance. EEG recordings of patients and controls showed different microstate features. More importantly, when differentiating among patients and controls, EEG microstate features outperformed conventional EEG ones. The performance of the microstate features exceeded that of conventional EEG, even after optimization using recursive feature elimination. EEG microstate features applied with conventional EEG features also showed better classification performance than conventional EEG features alone. The current study is the first to validate the use of microstate features to discriminate schizophrenia, suggesting that EEG microstate features are useful for schizophrenia classification.

## Introduction

Schizophrenia is a mental illness whose fundamental nature is not fully understood. Although Emil Kraepelin [1] and Eugen Bleuler [2] developed and advocated conceptualizations of schizophrenia, they alone do not explain its various manifestations. Several studies since then [3, 4] have documented changes in cognitive function as core symptoms of schizophrenia, and with advances in neuroscience modalities, many researchers have attempted to reveal clinical symptoms of schizophrenia objectively. Their work has improved understanding of the illness, thereby enabling causes to be identified and treatments to be developed. Nonetheless, objective research of schizophrenia continues to be very important.

Electroencephalography (EEG) measures electrical activity of the brain and is one of the most widely used modalities in schizophrenia research. EEG can analyze neural synchrony associated with the pathophysiology of schizophrenia [5]. EEG-measured neural activity reflects self-generated oscillation and large-scale synchronization with responses to external information [6]. Moreover, EEG present excellent time resolution. Thus, it is suitable for studying complex cognitive functions. Therefore, EEG can provide unique information that is otherwise difficult to obtain using imaging modalities.

Many features derived from EEG were exploited for schizophrenia classification. The power spectra has long been the most widely used feature [7–10] and has been applied to other psychotic disorders as well [11]. Johannesen et al. used frequency domain features in the working memory task to differentiate between patients with schizophrenia and healthy controls [12]. Schizophrenia classification was also performed using statistical features including mean, skewness, and kurtosis [13, 14]. It has been reported that the statistical descriptor of the variability within the EEG signal, the entropy measure and the fractal dimension related with it, are useful for schizophrenia classification [15–18]. In addition, candidate features that may be applied to schizophrenia classification was exploited in other research area [19, 20]. Unfortunately, no “gold standard” has been established for analyzing EEG data to diagnose and treat schizophrenia. There is no clear definition of the pathological levels, and, in terms of clinical EEG interpretation, intra- and interrater reliabilities have proven insufficient [21] compared to other diagnostic tools. Although many researchers have proposed various features for schizophrenia classification, such features do not resolve inconsistencies or ambiguities in EEG interpretations. Furthermore, such features have failed to utilize the greatest advantage of EEG: its millisecond temporal resolution. Therefore, it is important to develop diagnostic features that can exploit it.

Several attempts have been made to use EEG microstate analysis to overcome this limitation. For example, by segmenting spontaneous EEG at the sub-second level, Lehmann et al. (1987) demonstrated the existence of a quasi-stable microstate, producing stable and evenly patterned results at 80–120 ms intervals [22]. We can also assume that EEG captures the manifestations and changes of such microstates.

Many previous studies have revealed microstate changes in various diseases and mental states as “atoms of thought” [23] or “building blocks of mentation” [24]. Characteristic alterations in EEG microstates have been reported in tasks involving specific cognitive functions and sensory inputs [25] and in tasks requiring abstract reasoning [26]. Yuan et al. (2012) reported the association between microstates and known large scale networks using functional magnetic resonance imaging (fMRI) [27]. Studies have supported this association structurally [28, 29] and functionally [30]. Microstate analysis was performed under several psychiatric conditions (e.g., sleep [31], anxiety disorder [32], mood disorder [33], and neurodegenerative disorder [34–36]. In particular, the characteristic microstate change has been investigated [8, 24, 37–44] and the change in microstates C and D has been well-known [45–47] in schizophrenia. These studies have been performed in various areas related to schizophrenia, such as clinical symptoms [38, 39, 43, 48], genetic vulnerability [40], and medication [42] as well as presence of disease [24, 36, 37, 44]. Recently, the potential as a state and trait biomarker has been reported that can suggest the progress [49] and genetic underpinnings [41] of illness. Although clustering algorithms and methodological choices for microstate analysis are diverse [50, 51], EEG analysis method using a cluster of k-means proposed by Pascual-Marqui et al. [52] is widely used in schizophrenia researches. It was exploited in early microstate study [37] and provided four archetype microstates based on study with a large sample of subjects [53]. This method enables the use of four archetype microstates with high interpretability, where functional significance has been revealed based on resting-state fMRI networks [27, 30]. The functions of the four type microstates (i.e., A, B, C, and D) are known to be associated with auditory, visual, default mode, and dorsal attention, respectively [25, 27]. The features based on those microstates show differences between patients with schizophrenia and other groups and allow interpretation from a neuroscience perspective. Therefore, four archetype microstates were used not only in schizophrenia [24, 37–39, 46] but also in general medical conditions, such as physical exercise [54], insomnia [55], hearing loss [56]. In summary, the microstate analysis method using the four archetypes is suitable for schizophrenia research.

Although there have been microstate feature-based studies on schizophrenia, they were implemented using univariate, instead of multivariate, analysis. Univariate analysis is useful for validating hypotheses according to each variable. However, it does not fully exploit microstate features for building a classification or treatment model. Univariate analysis is a special case of the multivariate model that is straightforward and neatly structured, whereas multivariate analysis applies to complete or general cases [57]. With multivariate analysis, we can simultaneously analyze multiple dependent and independent variables to improve reliability and validity. Therefore, multivariate analysis can utilize all microstate-feature information and identify new patterns to improve understanding [58, 59]. Applying multivariate analysis to the microstate feature, we can create a more powerful model to reveal differences that are not detected in univariate analysis. Models having improved reliability and validity possess more advantages of interpreting and generalizing results. Machine-learning techniques (e.g., classification using kernel method) accomplish multivariate analyses that catalog distinct observations and allocate new observations to previously defined groups [60]. Thus, by applying machine-learning-based algorithms to microstate features, we can distinguish between EEG recordings of patients diagnosed with schizophrenia and those of healthy (control) subjects and present a practical application. In summary, machine-learning-based microstate analysis is a novel approach to schizophrenia classification.

Considering the characteristics of schizophrenia and microstate analysis, we hypothesize that multivariate microstate features based on four microstate archetypes are useful for schizophrenia classification. We first obtain microstate and conventional EEG features from recordings of patients diagnosed with schizophrenia and those of healthy (control) subjects. Next, we obtain classification accuracy for differentiating between diagnosed patients and controls using three sets of features: microstate, conventional EEG, and combined. Finally, we compare performances of the three features sets.

## Materials and methods

### Dataset

A publicly accessible EEG dataset was used in our study [61]. Study protocol was approved by the Ethics Committee of the Institute of Psychiatry and Neurology in Warsaw. All participants received a written description of the protocol and provided written consent to take part in this study. The dataset was obtained from 14 patients diagnosed with schizophrenia and those from 14 healthy (control) subjects. The patients’ group comprised seven males (27.9±3.3 years) and seven females (28.3±4.1 years) who were diagnosed with paranoid schizophrenia according to the International Classification of Diseases (ICD)-10-CM criteria F20.0 and who showed prominent positive symptoms. Other inclusion criteria were washout periods of more than 1 week. Early-stage patients, such as those exhibiting their first episodes, were excluded. Other exclusion criteria were as follows: pregnancy, organic brain pathology, severe neurological diseases (e.g. epilepsy, Alzheimer’s, or Parkinson disease), and presence of a general medical condition. Patient and control groups were matched by age and gender. Nineteen channel EEG were recorded in accordance with the International 10/20 EEG system with a sampling frequency of 250 Hz for 15 min each during an eyes-closed resting state. Detailed information about the dataset can be found at the repository and from its related article [61].

### EEG data preprocessing

**Fig 1:**
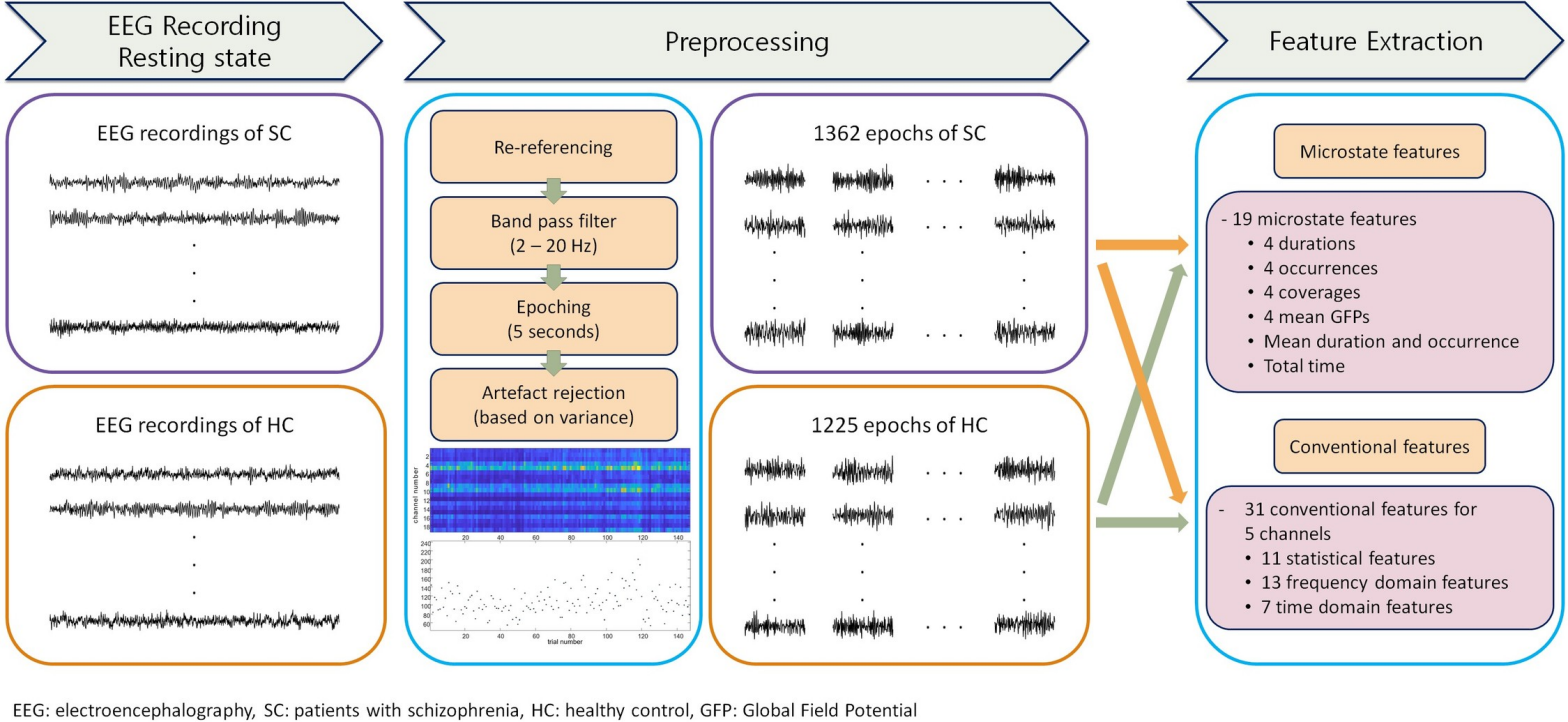
Overview of the process for extracting microstate and conventional EEG features.

To obtain artifact-free EEG features for microstate analyses, the EEG dataset was pre-processed using the FieldTrip toolbox and EEGLAB [62, 63]. We followed the well-established preprocessing pipeline that have been widely used in some previous studies [24, 25, 37, 53] to use the archetype microstates, which are proposed from the studies of schizophrenia and known for their functional significances. Fig 1 shows the entire process of extracting EEG features from raw data. EEG were re-referenced to the common average electrode and were filtered using a 2–20-Hz band-pass filter. Continuous EEG data were segmented into 5-s non-overlapping epochs evaluated based on variance, so that the epoch containing artifacts could be removed. From the continuous EEG dataset, we extracted 1,362 artifact-free epochs of patients diagnosed with schizophrenia and 1,225 artifact-free epochs of healthy (control) subjects. The benefit of this approach was that the all artifact-free data points could be analyzed instead of selecting and analyzing parts. All epochs were implemented for classification to distinguish whether each epoch was of the patients diagnosed with schizophrenia or healthy (control) subjects. To prevent the statistical power from being exaggerated in comparing the features between the two groups, the comparison was performed using only the first 20 artifact-free epochs as in previous studies [24, 36, 37, 53, 64]. Cohen’s D was reported with p-value to evaluate the effect size. The comparison performed with all epochs is presented in the S1 Table.

### Microstate analysis

#### Global Field Power (GFP)

GFP can be used to represent the global pattern of neuroelectrical activity and is defined as follows:

$$GFP(n)=\sqrt{(\sum_{i}^{N}{\left(x_{i}(n)-\bar{x}(n)\right)}^{2})/N}$$ (1)

where *x*_*i*_(*n*) and $\bar{x}(n)$ represent the instantaneous and mean potentials across N electrodes at time *n*.

Because GFP instantaneously measured electric-field activity, it was typically used to characterize the dynamic fluctuations of brain activity. Local GFP maxima described instances of the strongest brain potentials and the highest topographic signal-to-noise ratios. In microstate analysis, topographies of the electric field at local GFP maxima were discrete EEG states, and signal evolution was a series of such states [47]. Then, successive microstates, which were discrete states of the EEG analyzed based on local maxima of the GFP, were derived. Using clustering methods, all microstates can be assigned according to archetype microstates. The archetypes have enabled many studies which uncovered their function and applied them to various diseases [45]. In this study, modified k-means clustering were used for it. The clustering method were described in detail in the following sections and the parameters for microstate analysis can be found at http://www.thomaskoenig.ch.

Most microstate analysis studies have reported the same four archetypal microstate topographies to represent brain activity measured using a resting-state EEG. These four topographies included right-frontal left-posterior, left-frontal right-posterior, midline frontal-occipital, and midline frontal and are typed as A, B, C, and D, respectively [37, 53]. Importantly, single topography remained quasi-stable for intervals of about 80–120 ms before dynamically transitioning to another topography (i.e., microstates) [47]. Therefore, when an EEG was considered to be a series of topographies of electric potentials that evolve, the entire recording can be studied using a set of topography that dynamically fluctuate amongst themselves at discrete time points.

#### EEG microstate segmentation methods

Microstate analysis was used to segment entire EEG recordings into successive topographies, including spatiotemporal information of global brain network, that remained quasi-stable for a short period of time (80–120 ms). An early microstate analysis method proposed by Lehmann et al. segmented EEG signals using adaptive segmentation algorithms, wherein the topography at each successive GFP peak was compared to those at preceding ones, and a new microstate was considered when centroid locations of segmented potentials changed by more than a predefined interval [22]. Thus, the overall length and general topographic characteristics of microstates could be computed. However, early attempts employing adaptive segmentation rarely grouped microstates into archetypes.

More recently, Pascual-Marqui et al. [52] proposed a statistical approach that directly considered the topographies of entire EEG recording. This method utilized k-means clustering analysis, which iteratively combined nested high spatial-correlation topographies and identified a representative topography that best explained the variance in each cluster. Various methods of clustering, such as agglomerative hierarchical clustering [65], principal component analysis [66, 67], independent component analysis [68], a mixture of Gaussian algorithms [69], and Markov process-based decomposition [70, 71], recently developed for factor analysis, can be used to segment the most dominant spatial components in series of topography.

In this work, we used the modified k-means clustering algorithm to segment microstates obtained from EEG recordings [45] to determine the optimal sorting order of clusters using the cross-validation criterion. Previous well-established microstate studies using multi-modality data including EEG and resting-state fMRI [27, 30, 37, 53] have revealed that four topographies across all subjects were optimal to fully represent whole brain activity and allow interpretation from a neuroscience perspective. The four topographies included right-frontal left-posterior, left-frontal right-posterior, midline frontal-occipital, and midline frontal and are typed as A, B, C, and D, respectively. Therefore, in this work, we adopted these four most common microstate topographies A, B, C, and D as the number of optimal archetype templates. In addition, the sorting of microstates (A, B, C, D sorting) was performed using cross-validation strategies on the training EEG trials. The sorting order by k-means clustering, which led to the lowest free energy on training EEG inputs, is selected.

Once we determined the optimal number of microstates (four archetype microstates), next step is to sort and label them into a sequence by using modified k-means clustering algorithm and Global Explain Variance (GEV) criteria [65]. The setting parameters for k-means algorithms are re-run and iterations as explained following. In principle, by re-running the stochastic k-means algorithm multiple times (in this analysis, we set re-run parameter to 20 times), we are able to test multiple segmentations on the same dataset and select the best re-run based on the GEV criteria [65]. GEV is a measure of how similar each EEG sample is to the microstate prototype it has been assigned to. The higher the GEV the better [65]. More importantly, we are able to reach the global minimum among 20 local minimums (20 re-runs). After 20 re-runs, the one that maximizes the GEV is selected. However, the number of re-run is a trade-off between computation time and how likely we are to converge on the same optimal solution. In the Microstate EEGlab toolbox [50, 51] and its Python package in which we have applied for our analysis select 10 re-run as a default value. In addition, Thomas Koenig’s manual (http://www.thomaskoenig.ch) has recommended that a range from 20 to 50 re-runs could be sufficient for a proper analysis. Furthermore, we have found that there are several existing EEG microstate analysis literatures that set 10 re-runs [52] as well as papers that use 30 [26] as a proper re-run number.

Another parameter for k-means clustering is iteration, which means that in each re-run the k-means algorithm keeps iterating until some stopping criteria (convergence threshold) are satisfied. In this analysis, we used the convergence threshold, which stops the algorithms when the relative error change between subsequence iterations is below the threshold. Here, we set the threshold at 10^−6^. The maximum number of iterations set to 1000 which means the algorithm can stop if the maximum iteration is reached before convergence for computation time efficiency.

### Conventional EEG feature extraction

We compared classification performances obtained using microstate and conventional EEG features. First, electrodes were clustered into five regions of interest (ROI): left anterior (Fp1, F7, F3), right anterior (Fp2, F4, F8), left posterior (T7, C3, P7, P3, O1), right posterior (C4, T8, P4, P8, O2), and central (Fz, Cz, Pz). Then, conventional EEG features were extracted from each ROI. Based on numerous features extracted from previous studies [15, 20], 31 features suitable for resting-state EEG recordings were selected. A summary of all the defined features is presented in Table 1. The frequency band of the spectral analysis was defined as follows: δ (2–4 Hz), θ (4–8 Hz), α (8–13 Hz), and low-β (13–20 Hz). Finally, the analysis was performed using 155 features.

**Table 1:** Summary of conventional EEG features obtained for patients diagnosed with schizophrenia and healthy (control) subjects.

No.	Feature name	Abbreviation	Definition	Domain
1	Mean	μ	$\frac{1}{N}\sum_{n=1}^{N}x[n]$	Statistical
2	Variance	Var	$\frac{1}{N-1}\sum_{n=1}^{N}{\left|x[n]-\mu \right|}^{2}$	Statistical
3	Standard deviation	σ	$\sqrt{\frac{1}{N-1}\sum_{n=1}^{N}{\left|x[n]-\mu \right|}^{2}}$	Statistical
4	Skewness	Sk	$\frac{1}{N-1}\sum_{n=1}^{N}{\frac{\left(x[n]-\mu \right)}{\sigma }}^{3}$	Statistical
5	Kurtosis	Kur	$\frac{1}{N-1}\sum_{n=1}^{N}{\frac{\left(x[n]-\mu \right)}{\sigma }}^{4}$	Statistical
6	Zero crossing rate	zcr	$\frac{1}{N-1}\sum_{n=1}^{N-1}1_{R}(x[n]x[n+1])$	Statistical
7	Upper margin	Upp	$Pr[X\geq x]\geq \frac{95}{100}$	Statistical
8	Lower margin	Low	$\mathrm{Pr}\left[X\leq x\right]\leq \frac{5}{100}$	Statistical
9	Width	Wid	Upp−Low	Statistical
10	Asymmetry	Asy	(Upp+Low−2×Median)/(Upp−Low)	Statistical
11	Coefficient of variation	CV	$\frac{\sigma }{\mu }$	Statistical
12	Total power	*P*_*total*_	$\sum_{f}PSD(x)$	Frequency
13~16	Absolute band power	*P*_*band*_	$\sum_{f_{1}}^{f_{2}}PSD(x)$	Frequency
17~20	Mean band power	*P*_*average*_	$\frac{1}{M}\sum_{f_{1}}^{f_{2}}PSD(x)$	Frequency
21~24	Relative band Power	*P*_*relative*_	*P_band_/P_total_*	Frequency
25	Shannon entropy	*H*_*Sh*_	$-\sum_{i}P_{i}\log P_{i}$	Time
26	Sample entropy	SampEn	$-\log\frac{A}{B}$	Time
27	Approximate entropy	ApEn	Φ^*m*^(*r*)−Φ^*m*+1^(*r*)	Time
28	Permutation entropy	*PE*_*D*_	$-\sum_{i=1}^{D!}P_{i}\log P_{i}$	Time
29	Higuchi fractal dimension	HFD	ln(*L*(*k*))/ln(1/*k*)	Time
30	Katz fractal dimension	KFD	$\frac{\log_{10}\left(m\right)}{\log_{10}\left(d/L\right)+\log_{10}\left(m\right)}$	Time
31	Hjorth parameter	*A*_*x*_	$\frac{\sum_{n=1}^{N}{\left(x[n]-\mu \right)}^{2}}{N}$	Time

### Classification

To assess whether microstate features were appropriate for schizophrenia classification, we used several machine-learning algorithms to compare classification performances of microstate and conventional EEG features. We used tenfold cross-validation to assess classification accuracy. Considering high inter-subject variability of EEG, the fold was generated by subject label so that all epochs of one subject was included in the training or test set. Four parameters were used to evaluate classifier performance: accuracy, area under the curve (AUC), sensitivity, and specificity. Permutation tests were implemented to evaluate the competency of classifiers. In this study, we performed 1,000 repetitions to obtain a p-value to verify whether or not the observed accuracy was obtained by chance or whether it represented results. The above procedures were performed using MATLAB^®^ software (MathWorks, Inc., Natick, MA, USA).

The classifier was selected based on stability and simplicity among various known classifiers from a previous EEG study [72] and other biomedical engineering research [73, 74]: the support vector machine (SVM) [75], Linear Discriminant Analysis [76], Naïve Bayes (NB) [77], random forest (RF) [78], and the k-nearest neighbor (KNN) [79]. To compare classification performances of microstate and conventional EEG features, we applied all classifiers to combined features to obtain accuracy.

#### Feature selection

In the neuroimaging machine-learning community, it was widely known that feature selection was an important step required prior to training classifiers [80]. Feature selection was essential, because it was used to select the most informative features and to discard the noise and artifacts, which helped enhance the classification performance and reduce complex computations and overfitting problems. In this work, we employed univariate t-tests and multivariate recursive feature elimination (RFE) as feature selection techniques. Univariate t-tests were performed on individual features to identify significant differences between groups, whereas multivariate RFE was used to investigate mutual relationships between multiple features.

#### Univariate feature selection

Univariate statistical t-tests have been used in many neuroimaging studies to show abnormalities in average signals for one or more brain features in an illness group compared to normal average signals for those in a healthy group. Recent discrimination studies have used such t-tests to select the most informative features for machine learning in neuroimaging [80, 81]. The key results of statistical test-based analyses were usually expressed as p-values. Subsequently, the optimal p-value cut-off for selecting relevant features was determined via cross-validation, and the features selected were used in the subsequent machine-learning analysis. In this work, we applied t-test-based feature selection techniques to machine-learning-based schizophrenia classification. Using t-tests on training data, we generated a result that required retaining only those features presenting significant changes in any of the feature measures (i.e., microstate measures) between the two groups at threshold p-values (p<0.05, p<0.01, p<0.005, and p<0.001). Bonferroni correction was applied for multiple comparisons.

#### Recursive feature elimination

Although the univariate t-test did not consider interactions between multiple patterns, RFE was a multivariate wrapper-based feature selection algorithm that ranked features based on their effects on classification [82]. The RFE ranking procedure was closely related to that of the SVM model. In each RFE iteration, an SVM model was trained. The lowest-ranking features were then removed, because they have the least effect on classification accuracy, whereas the remaining features were used for the SVM model in the next iteration. The sequential process was repeated until all features have been discarded. The features were then ranked based on the elimination sequence. A detailed explanation of the application of RFE algorithms to neuroimaging can be found in previous studies [80, 82]. In this work, we implemented a univariate t-test/multivariate RFE hybrid feature selection technique.

### Statistical analysis

The microstate topographies between groups were compared using topographic ANOVA (TANOVA) in the Ragu software [83]. TANOVA was a non-parametric randomization test that computed statistical differences using global field power of difference topographies [84]. Similarity was assessed for each microstate type as in the method of Koenig et al. [37] and Grieder et al. [64]. Microstate features were analyzed using independent t-tests. To prevent type-Ⅰ errors caused by the multiple-comparison problem, we evaluated significance using Bonferroni correction. Paired t-tests were performed to assess the significance of classification results obtained from the three feature sets. Four p-values (p<0.05, p<0.01, p<0.005, and p<0.001) were used to select univariate features. Statistical analyses were performed using MATLAB^®^ software.

## Results

### Microstate analysis

**Fig 2:**
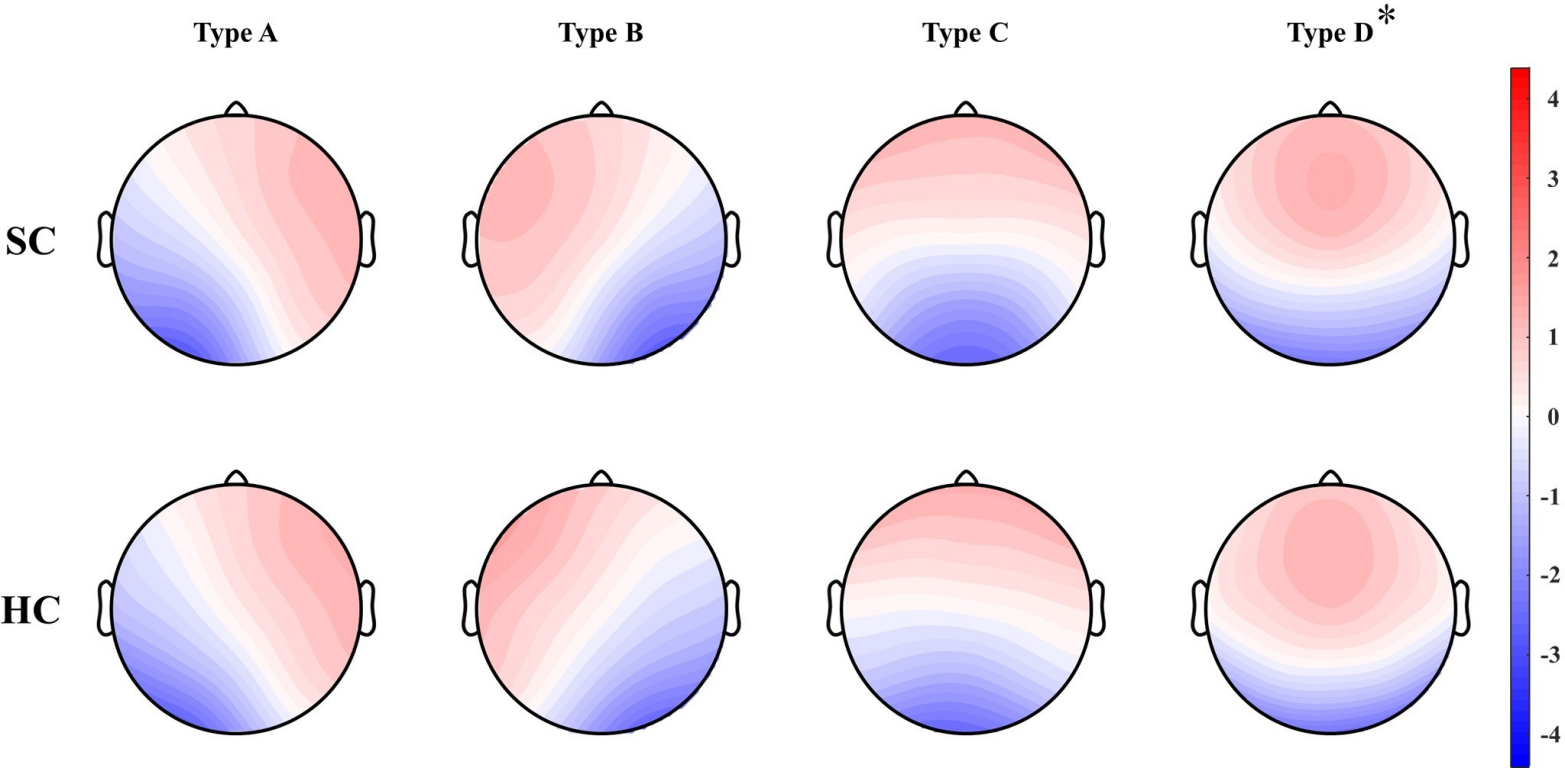
Four microstates of resting-state EEG recordings obtained for patients diagnosed with schizophrenia and healthy (control) subjects. Type D topography was significantly different between the two groups even after Bonferroni correction (p = 0.0012). Asterisk (*) show a significant difference. SC: patients diagnosed with schizophrenia, HC: healthy control.

Normalized microstate scalp topographies of a patient diagnosed with schizophrenia and a healthy (control) subject are shown in Fig 2. Types A and B were dominant in the unilateral frontal area, type C was symmetrical, and type D was somewhat dominant in the occipital area. These four microstates were similar to those shown in previous studies [37, 53]. Only type D topography showed significant difference between the two groups after Bonferroni correction. This was also consistent with the previous result [37]. Thus, the same archetype was applied.

Results obtained from microstate analysis conducted using 19 microstate features are presented in Table 2. There was no difference in total time between the two groups after Bonferroni correction. For types B and C in patients diagnosed with schizophrenia, coverage increased with increasing duration or occurrence. For type D in patients diagnosed with schizophrenia, coverage decreased with decreasing duration and occurrence. On the other hand, all type A features did not differ between patients diagnosed with schizophrenia and healthy (control) subjects. For across type features, there was no difference between the two groups in both mean duration and mean occurrence after Bonferroni correction. Although there was a difference in mean global field power (GFP) for types B, there was no difference for types A, C, and D. Among the significant features after Bonferroni correction, none of the features showed a small effect with Cohen’s d less than 0.2. On the contrary, all features that were not significant showed small effects with Cohen’s d less than 0.3. To summarize, eight microstate features showed significant differences between patients diagnosed with schizophrenia and healthy (control) subjects.

**Table 2:** 19 microstate features obtained for patients diagnosed with schizophrenia and healthy (control) subjects.

	Type A	Type B	Type C	Type D	Across types
**Duration** (ms)					**Mean duration** (ms)
SC	70.8(±19.40)	69.8(±20.74)	86.9(±37.32)	79.5(±23.66)	79.1(±17.43)
HC	72.0(±21.20)	65.1(±18.87)	73.4(±22.15)	90.7(±30.84)	77.6(±16.52)
Cohen’s d	−0.06	0.24	0.44	−0.41	0.09
p-value	0.564	0.014	**< 0.001**	**< 0.001**	0.363
t-value	−0.578	2.457	4.514	−4.171	0.911
**Occurrence** (Hz)					**Mean occurrence** (Hz)
SC	2.97(±1.06)	2.87(±0.89)	3.64(±1.09)	3.72(±1.20)	13.21(±2.79)
HC	3.04(±0.92)	2.60(±0.92)	3.61(±0.91)	4.16(±0.94)	13.40(±2.54)
Cohen’s d	−0.07	0.31	0.04	−0.40	−0.07
p-value	0.460	**0.002**	0.714	**< 0.001**	0.463
t-value	−0.740	3.143	0.367	−4.121	−0.735
**Coverage** (%)					
SC	20.6(±7.94)	19.8(±7.72)	30.7(±11.73)	29.0(±9.92)	
HC	21.4(±7.64)	16.5(±6.37)	25.9(±8.04)	36.2(±9.19)	
Cohen’s d	−0.11	0.46	0.48	−0.76	
p-value	0.257	**< 0.001**	**< 0.001**	**< 0.001**	
t-value	−1.136	4.723	4.931	−7.757	
**Mean GFP** (μV)					**Total time** (s)
SC	4.89(±1.35)	4.90(±1.38)	5.16(±1.50)	5.22(±1.37)	4.551(±0.124)
HC	4.68(±1.82)	4.41(±1.60)	4.83(±1.86)	5.04(±1.91)	4.575(±0.087)
Cohen’s d	0.13	0.33	0.19	0.11	−0.22
p-value	0.170	**0.001**	0.048	0.270	0.022
t-value	1.374	3.356	1.986	1.105	−2.305

Statistical analyses were conducted using independent t-tests. After Bonferroni correction for multiple comparisons, a significant correlation was bolded. SC: patients diagnosed with schizophrenia, HC: healthy control, GFP: global field power, mean (± standard deviation).

### EEG analysis using conventional EEG features

Using the conventional EEG features presented in Table 1, we investigated differences between patients diagnosed with schizophrenia and healthy (control) subjects. A total of 155 features were tested using post hoc analysis in five ROIs at significance levels 0.05, 0.01, 0.005, and 0.001. Differences were found in 42, 31, 22, and 14 features, respectively. Among the statistical features, mean, kurtosis, skewness, and coefficient of variation did not differ in all ROIs. 13 of 55 statistical features showed significant differences. Each frequency-domain feature showed significant differences in at least one ROI, and 19 of 65 features were different between groups. 10 of 35 time-domain features showed differences, and 3 of the 7 features did not differ significantly in all ROIs. Approximate and sample entropy features showed a very low correlation with schizophrenia. Overall, although some insignificant features were included, the conventional EEG feature set consisted of several features useful for schizophrenia classification.

### Classification performances obtained using microstate and conventional EEG features

To compare performances obtained using microstate and conventional EEG features in classifying patients diagnosed with schizophrenia and healthy (control) subjects, we applied several classifiers to each feature set. The classification accuracy obtained by tenfold cross-validation using several classifiers is presented in the S2 Table, and the result of classifier showing the highest classification accrual is presented in Table 3. The highest performance was 75.64%, obtained by applying the SVM quadratic kernel to microstate features. The conventional EEG feature also showed the highest average accuracy of 67.62% with the SVM. The highest accuracy with SVM was 72.93% when using microstate and conventional EEG features, which was significantly higher than when using only conventional EEG ones. The performances of the other classifier were found in the S2 Table. In summary, in the schizophrenia classification, the addition of microstate features to conventional features increased the accuracy of classification.

**Table 3:** Classification accuracies with quadratic SVM classifier using 19 microstate and 155 conventional EEG features obtained from the EEG dataset for patients diagnosed with schizophrenia and healthy (control) subjects.

Feature set	Accuracy (%)	AUC	Sensitivity (%)	Specificity (%)
Conventional EEG features	67.62	0.7292	64.47	69.16
Microstate features	75.64*	0.8019	71.93	75.50
Conventional + Microstate features	72.93*	0.7963	72.19	73.30

It is derived by SVM quadratic kernel, which shows the highest classification accuracy, and results of other 8 classifiers are shown in the S2 Table. Asterisk (*) shows a significant difference in comparison with accuracy using conventional EEG features (p< 0.05). AUC: area under the curve.

For accuracy calculated by tenfold cross-validation, microstate features showed higher accuracy than conventional EEG with NB, RF, KNN, and SVM classifers. The model of the highest accuracy is shown in Table 4 as the quadratic-kernel SVM using the microstate feature. The accuracy of each fold was evaluated using a permutation test with 1,000 repetitions. It was statistically significant in all folds.

**Table 4:** The classification results of tenfold cross-validation with quadratic SVM classifier using 19 microstate features.

Fold	Train Accuracy (%)	Test Accuracy (%)	Permutation test p-value	AUC	Sensitivity (%)	Specificity (%)
1	82.01	76.83	< 0.001	0.8195	75.66	78.29
2	83.54	74.60	< 0.001	0.7898	59.21	81.40
3	81.52	89.30	< 0.001	0.9579	83.51	92.53
4	83.55	63.81	< 0.001	0.6289	59.69	70.37
5	81.77	86.59	< 0.001	0.8416	71.43	90.28
6	82.31	81.78	< 0.001	0.8841	90.97	66.30
7	83.76	65.23	< 0.001	0.7710	59.30	76.70
8	81.85	81.09	< 0.001	0.8708	66.23	90.32
9	82.29	76.05	< 0.001	0.7698	85.88	51.47
10	84.53	61.14	< 0.001	0.6859	67.42	57.34
mean (± SD)	82.71	75.64 (±9.63)		0.8019 (±0.096)	71.93 (±11.69)	75.50 (±14.10)

SD: standard deviation, AUC: area under the curve.

### Classification performances with feature selection methods

Table 5 shows whether the superiority of microstate features was maintained after the feature selection method was applied. The results of the other classifier were listed in the S3 Table. For the microstate feature, only four features were removed: total time, duration, and coverage of type A and the coverage of type B. For conventional EEG features, including numerous insignificant ones, the highest accuracy was achieved when only RFE was applied. After feature selection was applied, the highest accuracies of the microstate and conventional EEG feature sets were 76.62 and 68.89%, respectively. Conventional feature set showed the highest classification accuracy using 136 features by removing 19 features with RFE. The highest classification accuracy of combined feature set was 76.85%, which was higher than that of conventional EEG ones alone. It was obtained using 39 features by removing 133 features with univariate feature selection and 2 features with RFE. Even in a subset of features showing the highest classification accuracy, microstate features outperformed conventional EEG ones. However, the addition of microstate features helped improve classification accuracy.

**Table 5:** Classification accuracies with quadratic SVM classifier achieved for different methods of selecting features from an EEG dataset for patients diagnosed with schizophrenia and healthy (control) subjects.

Feature set + (UFS threshold) + RFE	Before FS	RFE only	(p<0.001) + RFE	(p<0.005) + RFE	(p<0.01) + RFE	(p<0.05) + RFE
Conventional EEG features	67.62	**68.89**	66.83	67.13	66.26	67.79
Microstate features	75.64	**76.62** *	-	-	-	-
Conventional + Microstate features	72.93 *	74.31	76.00 *	**76.85** *	75.75 *	75.61 *

Classifier accuracy is presented as a percentage. The highest classifier accuracy in each set is bolded.

* Significant at p < 0.05 in a paired t-test compared to accuracy using conventional microstate features obtained using RFE. FS: feature selection, UFS: univariate feature selection, RFE: recursive feature elimination.

## Discussion

This study investigates the usefulness of EEG microstate features for schizophrenia classification. To validate microstate features, we first used a modified k-means method to extract them from EEG recordings of patients diagnosed with schizophrenia and those from healthy (control) subjects. Considering research reproducibility, we utilized all but some of the epochs, which consist of the remaining epochs after the epochs containing artefact were removed in the preprocessing step. Next, we applied feature selection methods to conventional EEG features introduced in a previous study [7–20] to compare classification performances. The method would prevent classification performance of conventional EEG features from being underestimated in multivariate analysis. Finally, after classification performances were obtained using microstate features, conventional EEG features, and combined features with tenfold cross-validation, average accuracies were compared. It showed higher classification accuracy than when using only convention features, both when only using the microstate feature and when combining microstate and convention features. Therefore, the results of this study suggest that microstate features are useful for distinguishing between schizophrenic and healthy (control) subjects. Combining microstate features with conventional EEG features is, thus, a proper application that can achieve higher classification accuracy than using only conventional ones.

The results of the microstate analysis, represented in Fig 2. and Table 2, showed characteristic changes in features for patients diagnosed with schizophrenia. The topography of type D showed a significant difference between the two groups, and the others did not. While this is consistent with previous schizophrenia studies [37], it differs from the result of semantic dementia and Alzheimer’s disease studies, which differed in types B and C [64]. Changes of duration and occurrence led to changes in coverage, mean duration, and mean occurrence. For patients diagnosed with schizophrenia, duration increased for type C and decreased for type D. According to Milz et al., each microstate can be associated with a specific function [30], and several features of the four types may change in each illness group [45]. The changes in EEG recordings of types C and D microstate features are in line with those reported in previous studies [24, 36, 40, 44]. Specifically, the type-D microstate feature showed reduced duration when a subject experienced hallucinations [39]. In agreement with results of previous studies, duration was reduced in the group of patients diagnosed with paranoid schizophrenia who showed prominent positive symptoms in this study. Type B should be interpreted with caution, because, although coverage [36] and occurrence [44] were consistent with those shown in previous studies, duration decrerased in the previous study [24, 36] increased in our study. In contrast to earlier findings that showed no change in occurrence or reduced occurrence [24, 36, 44], occurrence and coverage of type A increased. However, the features of types A and B had smaller effect sizes considering Cohen’s d. Thus, it is difficult to accept that the features actually differed between schizophrenia and healthy (control) subjects. Type A and B features, known to be associated with a sensory task, do not present remarkable differences. Considering the function of each type [27, 30], it is natural that types C and D, attributed to default mode and dorsal attention, respectively [25, 27], are more reliable diagnostic features than type A and B features in schizophrenia classification. Results consistent with previous studies have established that microstate features are suitable for schizophrenia classification.

Several studies reported abnormal functional brain network in schizophrenia and other psychiatric disorders. For example, depressive disorders, including major depressive disorders, have been examined in comparison with schizophrenia. They are classified as different diseases [85], however, share risk factors and may show similar clinical manifestations in the early or severe stage of the disorder [86]. The dysregulation of left inferior parietal cortex [87] and decreased convergent and divergent network connectivity [88] were consistent in both disorders. Lydon-Staley and Bassett showed the similarity of reduced small-world properties and differences in regional activation on two disorders [89]. In some regions or networks, the difference in activation was demonstrated [90–92], and the opposite activation was identified [93–95]. Taken together, previous studies of functional networks revealed similar and different alterations depending on the nature or areas of the feature applied. Considering that microstate features are associated with the functional network, these may also show common and distinct changes in various psychiatric disorders. The development of various features that can fully exploit the advantages of temporal resolution enables the microstate feature to be a diagnostic biomarker reflecting functional brain network.

Machine-learning based multivariate analysis provides an opportunity to understand a system by analyzing many features simultaneously. Therefore, we can reduce type Ⅰ errors and obtain optimized models with multivariate analysis. In other words, features extracted from EEG recordings containing some information can be utilized with multivariate analysis to develop a model having higher reliability and validity. This is an important process in the classification of schizophrenia for which EEG microstate analysis is a valuable method. Several studies have already reported that EEG features are useful in emotion recognition [20], neurological disease [96], and schizophrenia classification [16, 97]. To validate performances of microstate features in schizophrenia classification, we examined performances of conventional EEG features for comparison. Our results suggest that microstate features reflect schizophrenia characteristics and show better classification performance than conventional EEG features. The microstate feature was not only used alone, it also showed better performance when combined with conventional EEG features. We can improve the classification accuracy by combining microstates with conventional features. In other words, we can infer that microstate features contain information that is difficult to obtain by traditional EEG analysis methods. We expect microstate analysis to be synergistic with conventional EEG analysis methods. Taken together, microstate features show potential for schizophrenia classification.

Prior studies have applied multivariate analysis to various EEG features to classify patients diagnosed with schizophrenia from healthy (control) subjects [16, 98–101]. Although classification accuracies obtained in those studies were quite high, they may not have been sufficient to generalize results. For example, some studies were conducted without validation [98, 99], and only some channels were selected in other studies [16, 101]. Another study only used EEG event-related potentials generated during a specific task [100]. In the present work, because accuracy was calculated by applying tenfold cross-validation to resting-state-EEG data, classification performance is reliable. Furthermore, although machine learning has been applied to EEG microstate features for patients diagnosed with neurodegenerative diseases [102] and neurological disorders [103], it has not been applied yet to schizophrenia microstate studies. Although machine learning of microstate features is an alternative to existing methods of classification, it will likely develop into a more robust method when combined with other neuroimaging modalities, such as fMRI [45]. Therefore, our research investigating the classification accuracy of microstate features is essential to verify the feasibility of microstate analysis in schizophrenia classification. Regarding the performance of the microstate features used in this study, our method resulted in accurate classification and can be applied to various areas of neuroscience and clinical fields.

The strength of this study is that it has more generalizability than previous studies. First, we minimized bias caused by data selection. In previous studies, EEG recordings shorter than 10 s were selected from those longer than a few minutes [37, 53] for microstate analysis, which may have affected results. We divided all data into epochs and used all EEG recordings from all subjects to obtain overall classification performance. Therefore, classification performance in this study showed generality, because it considered EEG data for all subjects. Second, we applied cross-validation. In particular, considering inter-subject variability of EEG data [104], we chose tenfold cross-validation to prevent EEG epochs from having the same subjects belonging to both training and testing datasets. Because of these two reasons, despite the application of RFE to many of the features referenced in the literature, classification performance of conventional EEG features was relatively low. On the contrary, microstate features showed better performance, although the methods were not applied in all previous studies. Our reproducible results are likely to be replicated in future studies, suggesting that microstate features are promising diagnostic features for schizophrenia classification.

Some study limitations must also be discussed. This study used publicly available data instead of data obtained from cohort studies, and the number of subjects was small. Although age and gender were matched, it was difficult to obtain enough data to represent the general population. Thus, the results of this study should be interpreted carefully. Therefore, classification performances of microstate features should be analyzed using data obtained from a larger cohort to improve classification accuracy and to develop an accurate model representing the general population. Our study was performed using modified k-means clustering only with a k value of 4. In general, higher classification accuracy can be achieved by using other clustering methods or a higher k value [25, 105]. Although our study was performed only with a k value of 4, it is reliable to show a sufficient explained variance of patients diagnosed with schizophrenia (76.4±4.4%) and those of healthy (control) subjects (75.8±5.8%). However, using a different k value in future research may contribute to improvement of classification accuracy. Furthermore, the 19 microstate features used in this study may not fully reflect information obtained from microstate analysis. Although the number of features was not small, the included information sometimes overlapped. For example, the duration can be calculated from the total time, coverage, and occurrence. Thus, only the 19 microstate features used in this study did not fully exploit the strength of microstate analysis. Nevertheless, because microstate features showed promising classification performance, it is very probable that classification performance would be improved by developing new microstate features.

## Conclusions

In this study, we provided evidence for usefulness of EEG microstate features in schizophrenia classification. When comparing classification accuracies obtained using microstate features, conventional EEG features, and combined features, microstate features and combined features outperformed conventional ones, suggesting that EEG microstate features are appropriate for schizophrenia classification.

## Supporting information

S1 Table 19 microstate features obtained for patients diagnosed with schizophrenia and healthy (control) subjects. (DOCX)

S2 Table Classification accuracies obtained using microstate and conventional EEG features obtained from the EEG dataset for patients diagnosed with schizophrenia and healthy (control) subjects. (DOCX)

S3 Table Classification accuracies achieved for different methods of selecting features from an EEG dataset for patients diagnosed with schizophrenia and healthy (control) subjects. (DOCX)
