# Supplementary material for: EEG microstate features for schizophrenia classification
Source: PLoS One. 2021 May 14;16(5):e0251842. doi: 10.1371/journal.pone.0251842 (PMC8121321; doi:10.1371/journal.pone.0251842)
Supplement: S1 Table — (DOCX) [file pone.0251842.s001.docx]

**S1 Table. 19 microstate features obtained for patients diagnosed with schizophrenia and healthy (control) subjects.**

|  | **Type A** | **Type B** | **Type C** | **Type D** | Across types |
| --- | --- | --- | --- | --- | --- |
| **Duration** (ms) |  |  |  |  | **Mean duration** (ms) |
| SC  HC  Cohen’s d  p-value  t-value  **Occurrence** (Hz)  SC  HC  Cohen’s d  p-value  t-value  **Coverage** (%)  SC  HC  Cohen’s d  p-value  t-value  **Mean GFP** (µV)  SC  HC  Cohen’s d  p-value  t-value | 68.6(±16.86)  69.8(±19.90)  −0.07  0.093  −1.678  2.80(±0.95)  2.99(±1.02)  −0.20  **< 0.001**  −4.963  19.1(±7.28)  20.4(±7.59)  −0.18  **< 0.001**  −4.631  4.81(±1.28)  4.70(±1.70)  0.07  0.072  1.797 | 70.0(±18.99)  64.3(±18.03)  0.31  **< 0.001**  7.790  2.77(±0.82)  2.61(±0.89)  0.19  **< 0.001**  4.825  19.3(±7.19)  16.5(±6.52)  0.40  **< 0.001**  10.190  4.82(±1.24)  4.47(±1.58)  0.24  **< 0.001**  6.214 | 91.8(±31.08)  74.0(±23.34)  0.64  **< 0.001**  16.252  3.82(±0.92)  3.63(±1.00)  0.20  **< 0.001**  4.987  34.2(±10.52)  26.0(±7.89)  0.87  **< 0.001**  22.005  5.16(±1.32)  4.87(±1.75)  0.19  **< 0.001**  4.796 | 77.9(±22.89)  91.4(±31.71)  −0.49  **< 0.001**  −12.500  3.59(±0.97)  4.23(±0.95)  −0.66  **< 0.001**  −16.865  27.5(±9.28)  37.0(±9.56)  −1.01  **< 0.001**  −25.696  5.14(±1.29)  5.12(±1.84)  0.01  0.754  0.314 | 79.4(±14.42)  77.6(±17.31)  0.12  **0.003**  2.923  **Mean occurrence** (Hz)  12.99(±2.27)  13.47(±2.70)  −0.19  **< 0.001**  −4.864  **Total time** (s)  4.567(±0.105)  4.558(±0.109)  0.07  0.049  1.969 |
