# Supplementary material for: EEG microstate features for schizophrenia classification
Source: PLoS One. 2021 May 14;16(5):e0251842. doi: 10.1371/journal.pone.0251842 (PMC8121321; doi:10.1371/journal.pone.0251842)
Supplement: S2 Table — (DOCX) [file pone.0251842.s002.docx]

**S2 Table. Classification accuracies obtained using microstate and conventional EEG features obtained from the EEG dataset for patients diagnosed with schizophrenia and healthy (control) subjects.**

|  | SVM*_rbf_* | SVM*_lin_* | SVM*_quad_* | LDA*_lin_* | NB | RF | KNN 1 | KNN 5 | KNN 10 |
| --- | --- | --- | --- | --- | --- | --- | --- | --- | --- |
| Conventional EEG features  Accuracy (%)  AUC  Sensitivity (%)  Specificity (%)  Microstate features  Accuracy (%)  AUC  Sensitivity (%)  Specificity (%)  Conventional + Microstate features  Accuracy (%)  AUC  Sensitivity (%)  Specificity (%) | 62.71  0.7184  59.46  64.86  72.89 *****  0.7608  69.08  71.90  70.14 *****  0.7956  69.85  69.71 | 63.25  0.6506  57.05  65.56  71.43 *****  0.7516  67.08  70.02  73.33 *****  0.7807  69.42  73.49 | **67.62**  **0.7292**  **64.47**  **69.16**  **75.64** *****  **0.8019**  **71.93**  **75.50**  **72.93** *****  **0.7963**  **72.19**  **73.30** | 63.55  0.6681  56.34  66.47  71.83 *****  0.7551  68.33  69.84  73.94 *****  0.7891  70.24  73.78 | 58.82  0.5403  52.79  59.54  73.19 *****  0.7512  67.94  73.51  61.16 *****  0.5843  54.79  61.97 | 51.02  0.5091  45.36  54.91  74.31 *****  0.7156  67.49  77.57  72.16 *****  0.7340  65.95  71.97 | 57.38  0.5803  53.01  61.54  65.81 *****  0.6367  61.13  64.91  63.58 *****  0.6306  58.47  65.07 | 58.48  0.6341  54.35  62.37  69.18 *****  0.7389  64.59  68.54  65.44 *****  0.6994  60.83  65.54 | 59.43  0.6487  55.07  62.45  70.23 *****  0.7491  65.73  69.81  66.69 *****  0.7142  62.30  66.30 |

Classifier accuracy is presented as a percentage. The highest classifier accuracy is bolded. Asterisk (*) show a significant difference in comparison with classifier accuracy using conventional EEG features (p< 0.05). AUC: area under the curve, SVM: support vector machine, rbf: radial basis function, lin: linear kernel, quad: quadratic kernel, KNN-n: k-nearest neighbours with k value of n.
