# Supplementary material for: EEG microstate features for schizophrenia classification
Source: PLoS One. 2021 May 14;16(5):e0251842. doi: 10.1371/journal.pone.0251842 (PMC8121321; doi:10.1371/journal.pone.0251842)
Supplement: S3 Table — (DOCX) [file pone.0251842.s003.docx]

**S3 Table. Classification accuracies achieved for different methods of selecting features from an EEG dataset for patients diagnosed with schizophrenia and healthy (control) subjects**

| Feature set  + (univariate FS) + RFE | SVM*_rbf_* | SVM*_lin_* | SVM*_quad_* | LDA*_lin_* | NB | RF | KNN 1 | KNN 5 | KNN 10 |
| --- | --- | --- | --- | --- | --- | --- | --- | --- | --- |
| Conventional features  + RFE  + (p<0.001) + RFE  + (p<0.005) + RFE  + (p<0.01) + RFE  + (p<0.05) + RFE  Microstate features  + RFE  Microstate + Conventional features  + RFE  + (p<0.001) + RFE  + (p<0.005) + RFE  + (p<0.01) + RFE  + (p<0.05) + RFE | 62.71  62.98  58.31  60.32  61.62  **63.59**  72.89 *  **73.53** *  70.14  70.26  **74.43** *  74.29 *  73.38 *  72.84 * | 63.25  64.84  66.35  66.50  66.45  **66.68**  71.43  **72.53**  73.33  74.43  75.45  75.93  **76.07**  75.16 | 67.62  **68.89**  66.83  67.13  66.26  67.79  75.64  **76.62** *  72.93  74.31  76.00 *  **76.85** *  75.75 *  75.60 * | 63.55  64.82  63.71  66.21  **66.64**  65.43  71.83  **72.08**  73.94  74.05  75.55  75.78  **75.94**  75.61 | 58.82  61.22  **66.06**  64.61  65.05  64.21  73.19  **73.36**  61.16  61.52  **69.07**  67.59  65.87  65.76 | 51.02  59.90  59.12  61.83  **63.85**  62.99  74.31  **74.91** *  72.16  72.16  **74.52**  73.79  72.41  72.06 | 57.38  59.76  57.10  **60.42**  58.53  60.17  **65.81**  65.72  63.58  64.50  **68.14** *  67.01  66.61  66.85 | 58.48  61.15  58.77  60.29  60.16  **61.62**  69.18  **69.88**  65.44  66.54  **70.99** *  70.45 *  68.83  69.82 | 59.43  61.81  59.26  61.06  60.81  **61.94**  70.23  **71.05**  66.69  67.28  **72.46** *  71.17  70.93  71.37 |

Classifier accuracy is presented as a percentage. The most significant classifier accuracy is bolded. * Significant at p < 0.05 in a paired t-test compared to bolded accuracy using conventional microstate features obtained using RFE. FS: feature selection, RFE: recursive feature elimination, SVM: support vector machine, rbf: radial basis function, lin: linear kernel, quad: quadratic kernel, KNN-n: k-nearest neighbours with k value of n.
